# Clinical trial characteristics and intervention profiles for mild cognitive impairment: a systematic analysis of WHO ICTRP registry

**DOI:** 10.3389/fphar.2026.1701064

**Published:** 2026-01-19

**Authors:** Yingxuan Sun, Fengwei Zhang, Yinghong Zhou, Yonggen Yuan, Mengjia Li, Ji Xu, Hongyong Deng

**Affiliations:** Science and Technology Information Center of Library, Shanghai University of Traditional Chinese Medicine, Shanghai, China

**Keywords:** clinical trialregistries, clinical trials, drug development, ICTRP, mild cognitive impairment

## Abstract

**Introduction:**

Mild cognitive impairment (MCI) is a cognitive decline syndrome that represents an intermediate stage between normal aging and dementia, with a high risk of progression to dementia. The World Health Organization’s International Clinical Trials Registry Platform (WHO ICTRP) is the world’s largest clinical trial registry, aggregating data from global registries. This study aimed to analyze MCI trials in the ICTRP to delineate the research landscape, key focus areas, and emerging trends in MCI to inform future intervention strategies.

**Methods:**

A systematic study was performed to analyze MCI clinical trials registered in the ICTRP up to December 2024. Records were deduplicated, and extracted variables included study design, phase, country, sponsor, sample size, intervention type, and outcomes. Dual independent extraction was performed, with third-party adjudication for discrepancies.

**Results:**

A total of 2,609 MCI trials were included, with 51.8% registered in the past five years. The United States led in the number of registrations among countries, whereas Asia topped the list among continents. Of all the trials, 2,064 were interventional trials, while 499 were observational trials. Predominant types of interventions were non-pharmacological therapies, including non-invasive brain stimulation (211), cognitive interventions (194), computer-assisted cognitive interventions (192), dietary interventions (187), and exercise interventions (184). In the realm of pharmacological therapies, chemical drugs were the most prevalent. Clinical trials were concentrated in Phase 0/II. Most trials were sponsored by universities. In addition, 60.1% of the trials included fewer than 100 participants, and data sharing occurred at a low level.

**Conclusion:**

MCI trial registrations have been increasing annually. The treatment of MCI focuses on non-pharmacological therapies, with most pharmacological therapy studies in the early stages of clinical trials. Notably, integrated cognitive training that combines traditional methods with computer-assisted technologies has emerged as a particularly promising area of research.

## 1 Introduction

As the world population ages, dementia is currently the seventh leading cause of death and one of the major causes of disability and dependency among older people globally (WHO, 2025a). According to WHO, around 57 million people were estimated to have dementia across the world in 2021, with nearly 10 million new cases emerging annually (WHO, 2025a). A new case of dementia emerges every 3 s (Alzheimer’s Disease International, 2024). In 2024, nearly 12 million family members and other unpaid caregivers provided an estimated 19.2 billion hours of care to individuals with dementias (Alzheimer’s Association, 2025). The costs associated with dementia are also expected to more than double from US$1.3 trillion per year in $2019 to $2.8 trillion dollars by 2030 (Alzheimer’s Disease International, 2023). While we have seen significant progress in the diagnosis and treatment of dementia in recent years, we are still far from finding a cure, and even further from having a healthcare system that can provide promising treatments to everyone in need.

MCI represents a transitional stage between healthy aging and dementia. It is a clinically syndrome characterized by cognitive decline that exceeds the expected level for an individual’s age and educational background, while not significantly impairing activities of daily living (Gauthier et al., 2006). MCI affects approximately 15% of community-dwelling individuals aged 50 years or older worldwide (Bai et al., 2022). Patients with MCI are at high risk of progressing to dementias within a relatively short period of time than age-matched controls (Petersen et al., 2018). Epidemiological studies show that the estimated annual progression rate of individuals with MCI converting to dementia is 10%–15%, which is approximately ten times higher than the baseline incidence of dementia in the general population of the same age (Eshkoor et al., 2015). “Prevention is better than cure”, MCI serves as a preclinical, transitional phase between normal aging and dementia, and represents what researchers and clinicians view as a “window” in which it may be possible to intervene and delay progression to dementia (Anderson, 2019). The prevention and management of MCI may help mitigate dementia progression while potentially reducing both dementia prevalence and associated treatment costs.

Clinical trials represent a vital methodology in medical research. The registration of clinical trials serves to mitigate various forms of research bias while preventing resource waste resulting from study duplication (Kimball and Weinstock, 2005). This practice significantly contributes to the advancement of evidence-based medicine and, consequently, promotes progress in global public health. A growing number of academic journals have established collaborative partnerships with clinical trial registry platforms, rendering trial registration an essential prerequisite for publication consideration in clinical research (De Angelis et al., 2004). In 2006, the World Health Organization (WHO) launched the International Clinical Trials Registry Platform (ICTRP) in response to World Health Assembly Resolution WHA58.22. This global initiative aggregates clinical trial data from participating registries worldwide, assigns unique identification numbers to each registered trial, and serves as a centralized search portal for international clinical research. As per the latest update accessed on May 2025, there 18 Primary Registries that comply with standardized registration requirements, and 20 Data providers who are responsible for a database that is used by one or more registries (WHO, 2025b). As of June 2019, ICTRP is recognized as the largest clinical trial platform (Juneja et al., 2019; Namiot et al., 2023).

The WHO ICTRP encompasses the most registered studies (including unpublished or ongoing studies (Banno et al., 2020), and those with negative or neutral outcomes (Fernández-González, 2023) in the world, thereby providing a more comprehensive overview of global research activity. Analysis of clinical trial registration data from the WHO ICTRP offers valuable insights into current research trends across various medical domains. Because these data become available well ahead of journal publication, they offer a valuable forward-looking perspective on the trajectory of scientific inquiry. The goal of this study is to describe the main characteristics of current clinical trials on MCI to help identify any important research gaps. This will provide an overall idea of the aims and designs of studies that could obtain results in the years to come. The information will be useful for researchers, policymakers, patients, and the public, ultimately contributing to better healthcare outcomes.

## 2 Materials and methods

### 2.1 Data acquisition

We conducted a comprehensive search of all clinical trials related to MCI registered in ICTRP from inception to 31 December 2024. The search was performed using the standard search interface of the WHO ICTRP (ICTRP, 2025), with no additional restrictions applied to the search parameters. The search strategy was developed including the following terms: “MCI” OR “mild cognitive impairment” OR “mild cognitive disorder” OR “mild cognitive decline”, with no additional restrictions applied to the search parameters. All data was imported into CSV format and stored in Microsoft Excel to facilitate further data selection, classification, and management.

To classify clinical trials according to their conditions, we utilized the MeSH (Medical Subject Headings) database. This comprehensive database contains common entry terms and categories for various medical conditions. By integrating the MeSH database’s categories with the Conditions listed in the ICTRP dataset, we constructed a novel Condition table that associates conditions with each trial’s ID. Other non-relevant categories were excluded from the dataset.

### 2.2 Data pre-processing

Data preparation is a critical step in analysis, as it directly determines the integrity and accuracy of results. Our data cleaning process began with the removal of duplicate entries. In the ICTRP platform, duplicates may occur when a single trial is registered through multiple registries. These duplicates fall into two categories: visible and hidden. Visible duplicates would be flagged and resolved by the platform itself. Hidden duplicates, however, are not explicitly marked and may remain undetected. Our procedure addressed both types to ensure data reliability (Namiot et al., 2024): 1) Visible duplicates were identified and removed using the system flags, specifically the “Bridged type” field. 2) Hidden duplicates were detected by matching records with identical target sample size, primary sponsor, and closely similar public and scientific. Among these potential duplicates, we retained the entry with the earliest enrollment date and the most complete dataset. When enrollment dates were identical, the trial with the ID in alphabetical order was selected.

### 2.3 Data extraction and analyses

The following information was collected from each study: title, sponsor, registration date, data source, recruitment status, sample size, trial type, study design, phase, country, intervention protocols, outcomes, resultant linked to registry. The overall status of registered clinical trials on MCI were reviewed and collated. Categorical data are presented as number (n) and percent (%). For drug categorization, it was based on differences in origin, structural complexity, and production methods, also referencing the drug classification systems used by the Food and Drug Administration (FDA), Japan’s Pharmaceuticals and Medical Devices Agency (PMDA), and China’s National Medical Products Administration (NMPA).

Two independent researchers (SYX and LMJ) manually reviewed the trial information. Disagreements in the data extraction were adjudicated by a third researcher (XJ).

## 3 Results

**FIGURE 1:**
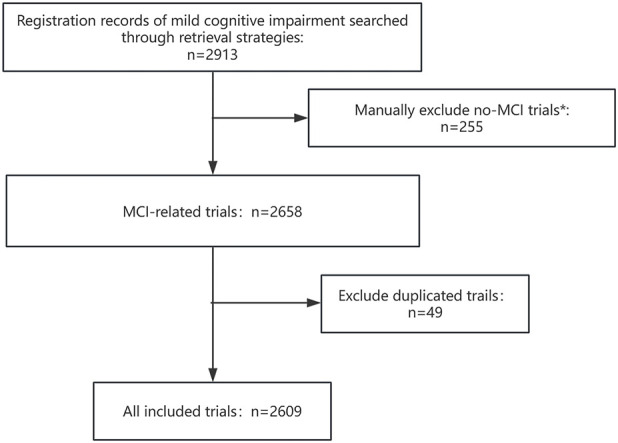
Flow chart of data search. * We consulted a geriatrician and used the Mesh vocabulary to review the MCI trial, identified those that focused on MCI diseases or conditions.

The systematic search identified 2,913 registered clinical trials in ICTRP platform as of 31 December 2024. 304 records were excluded after remove duplicates and manual screening, an additional 2,609 trials were ultimately retained for further analysis (Figure 1).

2,609 trials were sourced from 18 registries across 61 countries, with registration years spanning from 1999 to 2024. The collection comprised 2,064 interventional studies, 499 observational studies, and 46 other trial types. These studies encompassed a variety of allocation designs such as parallel, single-arm, crossover, factorial, sequential, and multi-arm. The primary sponsors were predominantly universities, hospitals, and industry or research institutes. The majority of clinical trials targeted a sample size of fewer than 100 participants.

### 3.1 Distribution of registration years and registries

**FIGURE 2:**
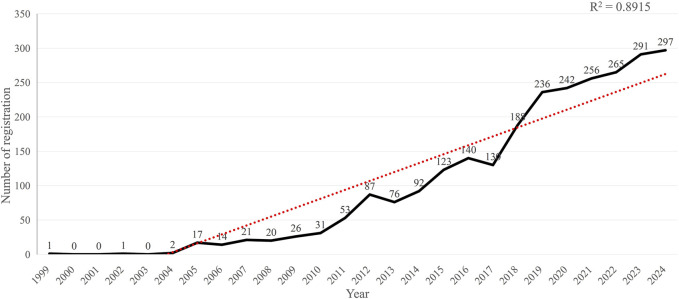
Number of registered MCI clinical trials in ICTRP.

A total of 2,609 MCI trials were registered during the period of 1999–2024. We constructed a graph of the number of registrations (Figure 2) using Microsoft Excel. There was a steady-jump-leap growth trend in the number of registrations, from one trial in 1999, 17 trials in 2005, 87 trials in 2012, and 297 trials in 2024. The annual number of registrations was less than 2 from 1999 to 2004. A steady increase began in 2005, with annual registrations first exceeding 100 trials in 2015. Although fluctuations occurred during 2013–2017, the overall trajectory showed consistent growth. A marked acceleration commenced in 2019, and the annual registrations exceeded 200 from then on. This demonstrated growing global recognition of MCI research importance within the scientific community.

**FIGURE 3:**
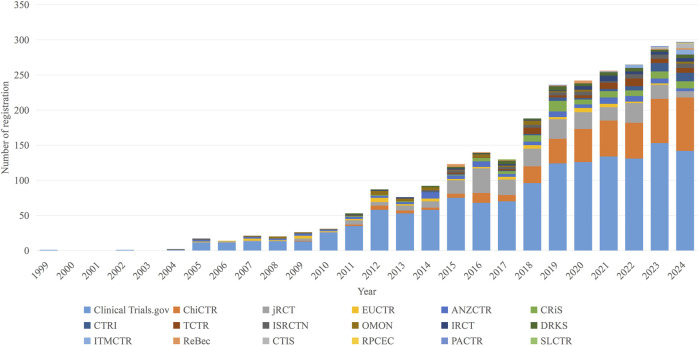
The annual number of Mild Cognitive Impairment (MCI) clinical trials registered in the registry center.

The clinical trial data were sourced from 18 registries. ClinicalTrials.gov was the most productive data providers (1,412, 54.1%), followed by Chinese Clinical Trial Registry (ChiCTR) (392, 15.0%) and the Japan Registry of Clinical Trials (jRCT) (266, 10.2%). Other contributing registries included the following: the Australian New Zealand Clinical Trials Registry (ANZCTR) (92), the Clinical Research Information Service (CRiS) (81), the EU Clinical Trials Register (EU-CTR) (56), the Thai Clinical Trials Registry (TCTR) (54), the ISRCTN registry (ISRCTN) (46), the Clinical Trials Registry-India (CTRI) (45), the German Clinical Trials Register (DRKS) (43), the Netherlands Trial Register (OMON) (42), the Iranian Registry of Clinical Trials (IRCT) (37), the Brazilian Clinical Trials Registry (ReBec) (15), the International Traditional Medicine Clinical Trial Registry (ITMCTR) (12), the Clinical Trials Information System (CTIS) (11), the Pan African Clinical Trial Registry (PACTR) (3), the Cuban Public Registry of Clinical Trials (RPCEC) (1), and the Sri Lanka Clinical Trials Registry (SLCTR) (1). Figure 3 shows the annual registration trends across these registries per year from 1999 to 2024.

Collectively, the distribution of MCI-related trials across the registries was highly uneven. The top three registry centers contributed to 79.34% of all MCI-related trials. Whereas annual registrations at ClinicalTrials.gov and ChiCTR exhibited a sustained upward trend, jRCT registrations declined in recent years. A few registries such as PACTR, RPCEC and SLCTR recorded fewer than 10 MCI-related trials. Most registries began listing MCI trials after 2005; some registries including EUCTR, CRiS, and OMON recorded zero registrations in certain years.

### 3.2 Country/Regional distribution

Among the 2,609 MCI trials, 162 trials did not provide country information, while 100 trials from multinational collaborations. The other 2,347 trials were registered by 61 individual countries, with 24 countries registering more than 10 trials (Table 1). The United States, China, Japan were the top three countries in terms of the number of MCI registered trails. Asia was the continent with the highest number of MCI registration trials, accounting for nearly half of the total trials obtained. This indicated a high level of concern about MCI disease in Asia.

**TABLE 1:** Regional distribution of MCI clinical trials (n > 10).

Continent	Country	Number of registrations
Asia (1078)		
​	China	573
​	Japan	219
​	South Korea	123
​	India	45
​	Iran	38
​	Thailand	35
​	Israel	21
​	Singapore	14
​	Pakistan	10
North America (669)		
​	United States	596
​	Canada	73
Europe (378)		
​	Germany	65
​	Italy	55
​	United Kingdom	52
​	Netherlands	52
​	Spain	44
​	France	33
​	Switzerland	25
​	Sweden	15
​	Norway	14
​	Belgium	12
​	Turkey	11
Oceania (82)		
​	Australia	82
South America (27)		
​	Brazil	27

### 3.3 Characteristics of MCI clinical trial design

The analysis of the included trials provided comprehensive insights into sponsors, study types, and trial designs, as detailed in Table 2. Most clinical trials of MCI were sponsored by universities, hospitals, industries, research institutes, individual investigators, government. Public-academic and industry partnerships together cover the great majority of participants and fiscal volume. After excluding 20 trials with unspecified sponsorship, the distribution was as follows: universities sponsored the majority of trials (1,187, 45.50%), followed by hospitals (644, 24.68%), industries (320, 12.27%), research institutes (161, 6.17%), individual investigators (144, 5.52%), and government entities (133, 5.10%).

**TABLE 2:** Descriptive information of sponsor, study type and trial design.

Category	Specifics		Number of records	Percentage of records (%)
Primary sponsor				
	University		1187	45.50%
	Hospital		644	24.68%
	Industry		320	12.27%
	Research institute		161	6.17%
	Individual		144	5.52%
	Government		133	5.10%
	Not reported		20	0.77%
Type of study				
	Interventional study		2064	79.11%
	Observational study		499	19.13%
	Others		46	1.76%
Study design				
	Assignment			
		Parallel	1942	74.43%
		Single	206	7.90%
		Crossover	150	5.75%
		Factorial	61	2.34%
		Sequential	36	1.38%
		Multiple arm	6	0.23%
		Not reported	208	7.97%
	Method of allocation			
		Randomised	1328	50.90%
		Non-randomised	285	10.92%
		Not reported	996	38.18%
	Masking			
		Open trial	1202	46.07%
		Single blind	268	10.27%
		Double blind	304	11.65%
		Triple blind	117	4.48%
		Quadruple blind	130	4.98%
		Not reported	588	22.54%
	Enrollment			
		0–100	1568	60.10%
		100–500	786	30.13%
		500–1000	97	3.72%
		1001–5000	81	3.10%
		>5001	25	0.96%
		Not reported	52	1.99%
	Purpose			
		Treatment	825	31.62%
		Prevention	255	9.77%
		Supportive care	112	4.29%
		Diagnosis	92	3.53%
		Basic science	61	2.34%
		Health services research	27	1.03%
		Screening	23	0.88%
		Educational	5	0.19%
		Device feasibility	4	0.15%
		Natural history	3	0.11%
		Not reported	1202	46.07%
	Phases			
		0	171	6.55%
		I	111	4.25%
		I/II	33	1.26%
		II	226	8.66%
		II/III	27	1.03%
		III	75	2.87%
		III/IV	3	0.11%
		IV	62	2.38%
		Pilot study	36	1.38%
		N/A	1865	71.48%

#Including trials with other type of study, such as cause/relative factors study (2), diagnostic test study (13), epidemiological research (1), prevention study (3), relative factors research (10), health services research (3), screen (3), basic science (6), other and blank (5)

A total of 2,609 trials were included, 2064 were interventional trials and 499 were observational trials. For assignment type, the parallel design represented the most frequent trial methodology (1942, 74.43%), 206 (7.90%) had single group assignment, 150 (5.75%) had crossover assignment, 36 (1.38%) had sequential assignment, 61 (2.34%) had factorial assignment, 6 (0.23%) had Multiple Arm assignment and 208 did not provide information. For Method of allocation, randomized allocation methods employed in over half of studies (1328, 50.90%), 285 (10.92%) had non-randomized allocation and 996 did not provide information. For trial purposes, the primary trial purposes were categorized as treatment (825, 31.62%), prevention (255, 9.77%), and supportive care (112, 4.29%). Blinding is a critical methodological element in randomized controlled trials (RCTs), which can effectively control the bias across multiple trial phases and minimizes differential outcome assessment (Moustgaard, 2020). The implemented blinding strategies ranged from single-blind (participants only), double-blind (participants and investigators), triple-blind (participants, investigators, and outcome assessors), to quadruple-blind (participants, care providers, investigators, and outcome assessors). Among 399 pharmacological intervention studies, double-blind (96, 24.06%) and quadruple-blind (90, 22.56%) were the predominant methods, whereas single-blind was rarely used (7, 1.75%). Among 1,344 non-pharmacological intervention studies, single-blind was the most common approach (261, 19.42%), followed by double-blind (208, 15.48%) and quadruple-blind (40, 2.98%). Clinical trial phases were concentrated in phases 0-II, with phase II representing the largest proportion. Approximately 60.10% of the registered clinical trials targeted a sample size of fewer than 100 participants.

**FIGURE 4:**
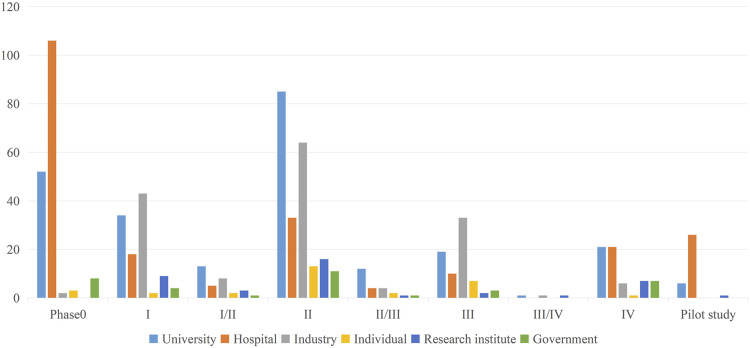
Distribution of sponsors across clinical trial phases for MCI.

Figure 4 indicated distribution of sponsors across clinical trial phases for MCI during 1999 through 2024 in ICTRP. As a key link between preclinical trials and formal clinical trials, Phase 0 was the second largest clinical stage in terms of registration and was conducted by hospitals and universities. Trials funded by industry were more likely to be phase II or III trials.

**FIGURE 5:**
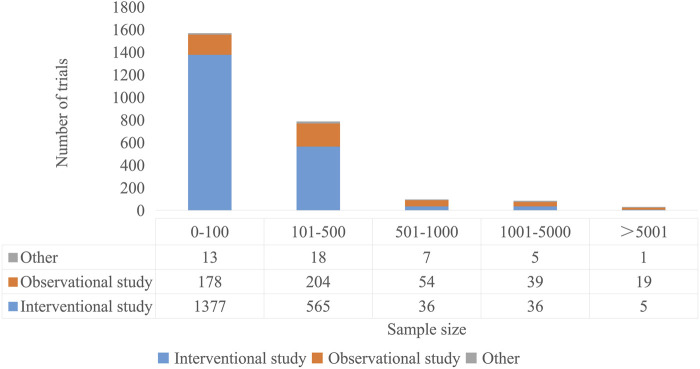
Distribution of sample sizes in MCI clinical trials.

Overall, the sample sizes of observational studies were larger than intervention studies (Figure 5). Specifically, intervention trials mainly had sample sizes ranging from 0 to 100 cases, while 40.88% of observational trials involved 101 to 500 cases (Figure 5). Observational studies common than intervention studies in all sample size intervals exceeding 500 cases. The largest recorded trial (NCT05796037) was an observational study with 1,500,000 participants which investigated chronic neurological diseases (including MCI, Alzheimer’s disease, Alzheimer’s dementia and Parkinson’s disease), it was sponsored by a company and registered in 2023.

### 3.4 Primary outcome measures

In the included trials, a total of 2,573 trials (98.62%) provided primary outcome measures, while 2,046 trials (78.42%) reported secondary outcome measures. The outcome measures of MCI trials mainly included the following aspects: a) Cognitive Function Assessment: Tools such as the Montreal Cognitive Assessment Scale (MoCA), Mini-Mental State Examination (MMSE), Alzheimer’s Disease Assessment Scale-Cognitive Subscale (ADAS-Cog), Auditory Verbal Learning Test (AVLT), Trail Making Test (TMT), Digit Span Forward and Backward (DSF/DSB), Boston Naming Test (BNT), and Verbal Fluency Test (VFT) were commonly used. b) Daily and Social Abilities Assessment: Scales including the Activities of Daily Living Scale (ADL), Instrumental Activities of Daily Living Scale (IADL), and Alzheimer’s Disease Cooperative Study-Activities of Daily Living Scale (ADCS-ADL) were employed. c) Psychological and Behavioral Symptoms: The Neuropsychiatric Inventory (NPI), Anxiety Scale, and Depression Scale were used to assess psychological and behavioral symptoms. d) Laboratory Tests: These included specific biomarkers such as plasma beta-amyloid protein (Aβ), p-tau protein, brain-derived neurotrophic factor (BDNF), serum extracellular vesicle miRNA. e) Brain Imaging Examination: Techniques such as functional near-infrared spectroscopy (fNIRS), brain magnetic resonance imaging (MRI), and electroencephalogram (EEG) signal acquisition were utilized.

The MMSE and MoCA scales emerged as the two most commonly utilized primary outcome measures, with 328 and 345 registered trials, respectively. These figures represented 12.57% and 13.22% of all primary outcome measures across the included trials. In clinical practice, MCI is predominantly assessed through neuropsychological testing, with the MMSE MoCA being the most extensively utilized instruments (Trzepacz et al., 2015). The MMSE is recognized as a relatively comprehensive and widely employed brief cognitive screening tool, suitable for thorough evaluation across various contexts (Arevalo-Rodriguez et al., 2021). Conversely, the MoCA is a freely accessible, concise cognitive screening tool available in multiple languages, specifically designed for repeated administrations (Horton et al., 2015). Conversely, tau protein and plasma beta-amyloid protein which hold significant importance in the diagnosis of Alzheimer’s disease (DeTure and Dickson, 2019) are also frequently employed as primary outcome measures in MCI clinical trials.

### 3.5 Interventions of MCI clinical trials

From the initial cohort of 2,609 MCI trials, we identified 2,061 intervention studies (79.11%) through meticulous type-specific screening. Following an exhaustive review of trial registration titles, study designs, and intervention protocols. We excluded trials that either (a) did not have MCI treatment or prevention as their primary objective or (b) employed undefined intervention methodologies. This process resulted in a final analytical dataset of 1,743 trials. These studies specifically examined therapeutic interventions aimed at improving or preventing MCI, which were categorized as pharmacological therapies (399 trials) and non-pharmacological interventions (1,344 trials).

#### 3.5.1 Pharmacological interventions

The 399 clinical trials focusing on pharmacological therapies as the primary intervention encompassed a diverse array of drugs, including many in the clinical development stage. According to the type of medication, it could be categorized into biological drugs, gene therapies (DNA/RNA-based), Chemical drugs, natural products and other types (Table 3). The majority of these therapies aimed to enhance cognitive function, mitigate memory loss, and improve quality of life, while others targeted concomitant symptoms such as sleep disturbances, dyslipidemia, hypertension, and depressive symptoms in patients with MCI. The drugs under investigation primarily included cholinesterase inhibitors, NMDA receptor antagonists (e.g., memantine), antidiabetic agents, gut microbiota modulators, melatonin, cardiovascular drugs, herbal preparations, and various novel therapeutics.

**TABLE 3:** Number of trails for agents with varying mechanisms of action.

Type of medication	Count
Chemical drugs	249
Natural product	79
Biological	62
Gene therapy (DNA/RNA-based)	5
Other*	4
Total	399

* Includes test products, unspecified drugs and drug codes that cannot be retrieved.

Among the most frequently evaluated drugs in these trials were donepezil (24 trials), insulin (12 trials), rivastigmine (6 trials), metformin (6 trials), melatonin (5 trials), and lecanemab (4 trials). Furthermore, several natural plant extracts were investigated, with Ginkgo biloba extract being featured in 14 trials and curcumin in 8 trials.

#### 3.5.2 Non-pharmacological interventions

The non-pharmacological therapeutic interventions examined in the clinical trials were categorized into the following groups: conventional cognitive interventions, non-invasive brain stimulation, computer-assisted cognitive interventions, dietary interventions, exercise interventions, traditional medical therapies, combined multimodal therapies and other interventions (Table 4). The distribution of these interventions was as follows (Lambez et al., 2020; Veronese et al., 2023): non-invasive brain stimulation (211 trials, 15.70%), conventional cognitive training (194 trials, 14.43%), computer-assisted cognitive interventions (192 trials, 14.29%), dietary interventions (187 trials, 13.91%), exercise interventions (184 trials, 13.69%), traditional medical therapies (70 trials, 5.21%), combined multimodal therapies (90 trials, 6.70%) and other interventions (216 trials, 16.07%).

**TABLE 4:** Distribution of non-pharmacological interventions in MCI clinical trials.

Category	Intervention	Number of registered clinical trials
Non-invasive brain stimulation (211)		
​	tDCS	65
​	TMS	61
​	tACS	49
​	Non-invasive brain stimulation	16
​	Neurofeedback	8
​	Intermittent theta-burst stimulation	8
​	Sensory stimulation	2
​	Intermittent limb pressure stimulation	1
​	Transcranial laser stimulation	1
Cognitive intervention (194)		
​	Cognitive training	103
​	Game-based training	38
​	Learning training	20
​	Memory training	17
​	Social skills training	9
​	Attentional process training	4
​	Reminiscence therapy	3
Computer-assisted cognitive intervention (192)		
​	AI	81
​	VR	50
​	Software applications	21
​	Cloud-based platforms	18
​	Digital therapeutics	15
​	Telerehabilitation systems	7
Dietary intervention (187)		
​	Dietary^a^	187
Exercise intervention (184)		
​	General exercise	148
​	Mind-body exercise	36
Traditional medical therapy (70)		
​	Acupuncture	59
​	Ayurveda	7
​	Therapeutic massage	4
Combined multimodal therapy (90)		
​	Cognitive training and physical exercise	36
​	Multidomain intervention	31
​	TMS & tDCS	6
​	tDCS & Tai Chi	5
​	tDCS & physical exercise	2
​	tDCS & VR	1
​	TMS & VR	1
​	TMS & Tai Chi	1
​	VR & Tai Chi	1
​	Therapeutic massage and aromatherapy	1
​	Electroacupuncture & AI-assisted therapy	1
​	Cognitive training and health education and music therapy	1
​	Cognitive training and dietary intervention	1
​	Tai Chi and dance therapy	1
​	tACS & acupuncture	1
Other intervention (216)		
​	Art therapy^b^	31
​	Medical care	26
​	Photobiomodulation therapy	25
​	Home-based care	22
​	Sleep intervention	14
​	Mindfulness training	13
​	Device-assisted therapy	12
​	Auditory intervention	11
​	Psychological intervention	10
​	Health education	9
​	Hyperbaric oxygen therapy	7
​	Aromatherapy	7
​	Continuous positive airway pressure	6
​	Oral intervention	5
​	Plasma exchange therapy	4
​	Olfactory intervention	3
​	Ophthalmic intervention	3
​	Unspecified method	2
​	Video-based therapy	2
​	Meditation	1
​	Whole-body cryotherapy	1
​	Catheter ablation for persistent atrial fibrillation	1
​	Nature-based therapy	1

^a^ Dietary therapy comprises specialized dietary regimens, therapeutic nutrition, vitamin supplementation, dietary supplements, and functional foods.

^b^ Art therapy encompasses music, poetry, calligraphy-based writing, and related modalities.

Among traditional medical therapies that included acupuncture, Ayurveda, and massage-acupuncture was the most frequently registered (59 trials). These trials encompassed traditional needling, electroacupuncture, and moxibustion. Non-invasive brain stimulation (NIBS) encompasses a range of neuromodulation techniques that utilize physical modalities (electrical, magnetic, optical, or ultrasonic) or chemical approaches to modulate neural activity in targeted brain regions without invasive procedures, thereby aiming to restore or maintain homeostasis within the nervous system (Begemann et al., 2020). The clinical trials employed multiple NIBS modalities, including transcranial direct current stimulation (tDCS), transcranial magnetic stimulation (TMS), transcranial alternating current stimulation (tACS), intermittent theta-burst stimulation (iTBS), transcranial laser stimulation (TLS), along with other unspecified NIBS methods. Among these, tDCS (65 trials), TMS (61 trials), and tACS (49 trials) emerged as the most frequently utilized techniques, demonstrating their predominant status in contemporary research applications. The study also systematically categorized cognitive training methods from the included trials into traditional cognitive interventions and computer-assisted cognitive interventions, reflecting the diversity of cognitive training strategies. Among traditional interventions, conventional cognitive training was the most prevalent (103 trials), followed by game-based training and medical care. Computer-assisted cognitive training was further categorized based on delivery medium into software-based, web platform-based, and other modalities. The distribution of these modalities included artificial intelligence (AI)-assisted training, virtual reality (VR)-based training, standalone software applications, cloud platforms, and telerehabilitation systems. These interventions primarily aimed to enhance memory, attention, processing speed, affective regulation, and communication skills in patients with MCI (Maggio et al., 2023). Dietary interventions (187 trials) included personalized nutritional plans, therapeutic diets, vitamin supplementation, dietary supplements, and functional foods tailored to patients’ physiological profiles. Exercise interventions comprised two main categories: General Exercise and Mind-Body Exercise. General Exercise includes aerobic, resistance, flexibility, and neuromotor training (e.g., cycling, strength training, and coordination drills). Mind-Body Exercise encompasses traditional practices such as Tai Chi, Baduanjin, and yoga. Amongst other unclassified therapies, art therapy, medical care for individuals with cognitive impairment, and photobiomodulation emerged as the three most prevalent approaches. Notably, combination therapies were frequently employed, with the integration of cognitive training and physical exercise being the most common multimodal intervention.

### 3.6 Subject recruitment and results availability

Among the 2,609 MCI trials in the study, 66.19% (1727) of them were labeled as “not recruiting”, 32.46% (847) as “recruiting”, and 0.84% (22) as “authorized”. The recruitment status of the remaining 0.50% (13) of clinical trials was not specified (Table 5). Out of these, 216 trials (8.28%) shared their findings through various dissemination methods, including registry documentation, online platform releases, articles in peer-reviewed journals, downloadable reports, data available upon telephone request, and unpublished sources. The availability of results was distributed as follows: registry documentation (147), journal articles (20), online platform publications (8), downloadable reports (6), unpublished data (34), and data requested via telephone (1).

**TABLE 5:** Recruitment and results of the MCI clinical trials.

Recruitment and results	Specifics	Number	Percentage of records (%)
Recruitment status (2,609)			
​	Authorised	22	0.84%
​	Recruiting	847	32.46%
​	Not recruiting	1727	66.19%
​	N/A	13	0.50%
Results url link (2,609)			
​	Reported	216	8.28%
​	Not reported	2,393	91.72%
Results sharing method (216)			
​	Registry documentation	147	68.06%
​	Unpublished results	34	15.74%
​	Published journal articles	20	9.26%
​	Online platform publication	8	3.70%
​	Directly downloadable reports	6	2.78%
​	Telephone-requested data	1	0.46%

## 4 Discussion

With the aging population, the prevalence of MCI among middle-aged and elderly people is increasing. Early identification and intervention of MCI are essential for delaying the progression to dementia. This paper performed a systematic descriptive analysis of mild cognitive impairment clinical trials registered with the WHO ICTRP during 1999–2024. The results demonstrated that detailed interrogation of ICTRP can provide insights into longitudinal trends in disease research.

In this study, we analyzed 2,609 clinical trials focused on MCI, which were distributed across 18 registration centers and involved 61 countries. Our results indicated that the number of MCI trial registrations has increased rapidly since 2005, with 97.2% of all trials being registered after this year. This surge corresponds with the 2005 proposal for mandatory trial registration. The Declaration of Helsinki mandates that clinical trials be registered in publicly accessible databases prior to participant recruitment. Furthermore, the International Committee of Medical Journal Editors (ICMJE) issued a mandate in July 2005, stipulating that member journals would only consider publishing results from trials registered in approved public registries (De Angelis et al., 2004). In response to the aging global population and increasing societal awareness of age-related health issues, the World Health Organization launched the Mental Health Gap Action Programme in 2008, prioritizing dementia as a key condition (Dal-Re et al., 2013). MCI is considered as a critical “window” of opportunity for intervention, with the potential to delay progression to dementia, thereby driving substantial research interest in this area. This was demonstrated by the 1,351 MCI clinical trials registered between 2020–2024, accounting for 51.8% of all MCI trial registrations over the past 5 years.

The majority of these trials were registered with the United States registry, (ClinicalTrials.gov), followed by the Chinese registry (ChiCTR), and the Japanese registry (jRCT). The primary regions for conducting MCI clinical trials were the United States, China, and Japan. Notably, Asia led in the number of MCI trials among all continents. This finding contrasts with other studies indicating a higher incidence of MCI among white Europeans (Sachdev et al., 2015). China and Japan rank among the top three countries for MCI trial registrations. This discrepancy may be attributed to variations in research investment, divergent trends in population aging, or differences in governmental policy priorities. The mismatch between the high prevalence and the relatively limited number of clinical trials in Europe may result from constrained investment in preventive research (Pistollato et al., 2025) and disparities in registration practices (WHO, 2019). European Union member states had predominantly funded late-stage pharmacological treatments for Alzheimer’s disease, leaving the prodromal MCI window severely under-resourced and likely curtailing the number of registered trials (Jakovljevic et al., 2024). Both China and Japan are among the top ten globally in terms of population size, experiencing negative population growth and facing the challenges of an aging population (Kondo, 2016; Tu et al., 2022). MCI is prevalent among the elderly, and the aging population has heightened the risk of MCI, thereby prompting China and Japan to intensify their focus on MCI research. Furthermore, proactive national policies in both countries have significantly promoted the advancement of relevant scientific studies (NHC, 2024; Yoshitaka, 2025).

Our statistical analysis indicated that interventional studies constitute the predominant proportion (79.11%) of clinical trials focused on MCI, treatments for MCI are primarily categorized into pharmacological and non-pharmacological interventions.

Within the study, chemical and biological drugs were the most commonly investigated. Consistent with our findings on trial characteristics, the primary pharmacological agents for MCI management comprise several therapeutic categories: 1) cholinesterase inhibitors (e.g., donepezil, rivastigmine, galantamine), which enhance cholinergic neurotransmission; 2) NMDA receptor antagonists (e.g., memantine); that mitigate excitotoxicity-mediated neuronal damage; 3) repurposed medications including antidiabetic agents, gut microbiota modulators, melatonin, and cardiovascular drugs; 4) herbal preparations; 5) monoclonal antibodies such as lecanemab and donanemab. Recent years have witnessed significant advancements in pharmacological interventions for MCI. Lecanemab and donanemab, approved by the FDA in 2023 and 2024 respectively, were monoclonal antibodies targeting β-amyloid that slow disease progression in patients with MCI (Nguyen et al., 2024). However, the high cost and side effects of these drugs limit their widespread clinical use. Meanwhile, there is increasing research on using herbal medicines to treat MCI. Natural product and traditional medicine such as Ginkgo biloba extract (Zhang et al., 2016), Curcumin extract (Dost et al., 2021), Yiqi Congming Tang (Shi et al., 2023), Jiannao Yizhi Capsule (Lian et al., 2009), and Liqiao Yizhi Tang (Ji et al., 2009) have been applied in MCI clinical practice. These applications are pivotal in assessing the efficacy of traditional Chinese medicine in treating MCI and enhancing the efficiency of developing novel MCI treatments. (Gu et al., 2021).

Current non-pharmacological approaches for MCI predominantly focus on improving cognitive function, including overall cognitive function, episodic memory, executive function, attention and processing speed. An analysis of the included studies indicates that non-pharmacological therapy has been a focal point of research as a treatment modality for MCI. These approaches have demonstrated clinically significant benefits, characterized by operational simplicity, cost-effectiveness, favorable safety profiles with minimal adverse effects, and improved patient outcomes (Horr et al., 2015). With technological advancements, particularly in computer technology and artificial intelligence, an increasing number of conventional cognitive training programs have been integrated with computer-based systems. The integration of advanced technologies has markedly enhanced the effectiveness of computerized cognitive function interventions. Additionally, advancements in virtual reality (VR) technology have significantly contributed to the field of cognitive function training (Qin et al., 2025; Yuan et al., 2025). Researchers (Kotsimpou et al., 2025) conducted a systematic review of 6 randomized controlled trials, demonstrating that computer-assisted cognitive interventions effectively enhance overall cognitive function, executive abilities, and memory in individuals with MCI. These interventions also mitigate psychobehavioral symptoms, such as depression. Despite the promise of computer interventions, they inherently restrict interpersonal social interactions, which are crucial in addressing cognitive decline in MCI patients. Consequently, computerized cognitive interventions should be utilized as adjunctive therapies and thoughtfully integrated with other intervention strategies to optimize their effectiveness.

In our study, dietary therapies accounted for 13.9% of the non-pharmacological intervention trials. Research on dietary therapies for MCI has predominantly adopted observational study designs. Systematic reviews have consistently shown that dietary modifications, including improved daily eating habits (Morris et al., 2015; McGrattan et al., 2018; Zhang et al., 2021), therapeutic diets, and various forms of physical activity (Karssemeijer et al., 2017; Huang et al., 2022), offer benefits for both the prevention and management of MCI. Key components of the Mediterranean diet, particularly extra virgin olive oil and plant-based foods such as brown rice, green tea, and avocado, have been demonstrated to exert significant positive effects across various cognitive domains, including executive function, processing speed, orientation, and attention. In contrast, animal-derived products like butter and cheese have been associated with detrimental impacts on cognitive performance (Gutierrez et al., 2021).

Research evidence indicates that both aerobic exercise (Huang et al., 2023) and resistance training (Huang et al., 2022) exhibit statistically significant efficacy in enhancing cognitive function among MCI. Furthermore, traditional mind-body exercises, particularly Tai Chi, Baduanjin and yoga, have shown considerable promise in improving cognitive function within MCI populations. These modalities confer beneficial effects on global cognition and memory domains through dual mechanisms involving physical conditioning and psychophysiological enhancement, thereby positioning them as viable exercise interventions for cognitive management in MCI (Song et al., 2022).

In addition, traditional medical therapies and non-invasive brain stimulation are frequently utilized as non-pharmacological interventions. Acupuncture, a fundamental component of traditional Chinese medicine (TCM), is based on core TCM principles. This method involves the tailored selection of acupoints and manipulation techniques according to MCI syndrome differentiation, with the aim of regulating the Governor Vessel, replenishing the marrow sea, opening brain orifices, and enhancing mental cognition (ZHU et al., 2015). Acupuncture has shown significant therapeutic efficacy for MCI. As its clinical application for MCI increases, treatment protocols have become more varied in terms of acupoint selection, manipulation techniques, and needle types. Non-invasive brain stimulation complements conventional rehabilitation therapy by employing safe, non-invasive methods to achieve neuromodulation through targeted cortical inhibition or activation (Kesikburun, 2022). The most commonly utilized stimulation modalities-tDCS, TMS, and tACS-have demonstrated efficacy in neural regulation and cognitive enhancement.

Recently, social work interventions (Yang et al., 2023) such as music therapy and play therapy have become gained prominence among non-pharmacological strategies for MCI. These interventions have been shown to yield favorable outcomes (Yang et al., 2022) and present distinct advantages for long-term application due to their operational simplicity, cost-effectiveness, and enhanced patient acceptability.

Sample size determination represents a critical methodological consideration in clinical trial design, it is estimated based on effect sizes, which may be derived from previous RCTs, meta-analyses, or pilot studies (Kunselman, 2024). An excessively large sample size can lead to the inefficient use of human, material, and financial resources and introduce unnecessary operational complexities. Conversely, an underpowered study with an insufficient number of participants compromises statistical reliability and increases the risk of bias (Lin, 2018). In this study, the sample sizes of MCI trials predominantly fell within the range of 0–100 participants, accounting for approximately 60% of all included trials. This distribution aligns with patterns noted in broader clinical trial registration studies (Gajendrareddy et al., 2014; Li et al., 2022) and may reflect the methodological characteristics of research in this field.

As a fundamental methodological safeguard in RCTs, proper blinding is essential for minimizing observer bias and participant expectations, thereby preserving the objectivity of results, enhancing internal validity, and ensuring study reproducibility (Schulz and Grimes, 2002). Our study analyzed the implementation of blinding separately for pharmacological and non-pharmacological interventions. Among pharmacological trials, double-blind and quadruple-blind designs were most commonly used, whereas single-blind procedures predominated in non-pharmacological trials. This difference in blinding practice is likely related to the type of intervention. Blinding is generally more feasible in pharmacological trials, whereas certain non-pharmacological interventions, such as manual therapies, often cannot achieve conventional double-blinding (D'Alessandro et al., 2022). Future research should address this issue by actively seeking methods to minimize bias. These may include strictly implementing outcome assessments by independent assessors who remain blinded, along with standardizing and monitoring treatment procedures to reduce interventionist bias. The appropriate application of blinding techniques can significantly enhance the quality of study design.

An analysis of the MCI trials registry data revealed that Phase II trials were the most prevalent, followed by Phase 0 studies. Phase II clinical trials represent the preliminary stage of therapeutic evaluation, aimed at assessing the initial efficacy and safety of treatments in target patient populations. The substantial proportion of Phase 0 studies suggests that MCI trials predominantly remain in early developmental stages. The number of Phase 0, I, and II trials significantly exceeded that of Phase III and IV studies. This showed that there were many novel drugs in the initial stages of development or undergoing efficacy and safety evaluations for MCI. However, a limited number of these drugs would obtain market approval in recent years. Among them, lecanemab has demonstrated some therapeutic efficacy, yet its notable adverse effects cannot be overlooked (Hossain et al., 2024). This further reflects both the difficulty in developing such treatments and the substantial, often unavoidable, risks involved. Collectively, these factors indicate that research on MCI remains in an early stage of exploration and validation.

Our analysis revealed that institutionally sponsored trials primarily conducted by universities and hospitals, constituted the majority of studies with sample sizes exceeding 500 participants. Universities, hospitals, served as the primary funding sources sponsor across all clinical trial phases, with comparatively limited contributions from industries. This discrepancy indicated that academic institutions played a crucial role in MCI research, bolstered by significant government funding, and highlighted the insufficient involvement of corporate entities during the initial research phase of MCI. Our analysis shows that early-phase trials still dominate clinical research. This may be because current early evidence is not strong enough to convince funders to invest heavily in confirmatory studies. Additionally, high development difficulty and low expected returns discourage corporate investment. Alzheimer’s disease drug development has a notably high failure rate, approximately 99% of trials showing no difference between drug and placebo (Cummings et al., 2019). Reflecting this difficult environment, some pharmaceutical companies like Pfizer have withdrew from early-stage neuroscience R&D (Mullard, 2018), while others including Eli Lilly, Merck, and Johnson & Johnson have stopped developing certain drug types such as β-secretase inhibitors (Tatulian, 2022).

The analysis of trial designs across all included studies identified deficiencies in the specification and completion of key indicators, suggesting inadequate completeness of trial registration information and suboptimal data reporting rates. Current recruitment status data showed that 32.46% of registered trials had progressed to the participant recruitment stage. Out of the 2,609 trials, 216 studies expressed a willingness to share data, 181 studies chose to store their raw data in clinical trial registries or public management platforms. For other ways, data could be obtained by contacting the researchers via email. However, this method significantly limited dataset availability. A study demonstrates that when researchers attempted to contact study authors via email, only 28% of the requested data were ultimately accessible (Corti et al., 2022). If authors do not proactively disclose their data, it becomes exceedingly difficult for other researchers to access it. Data sharing has the potential to reduce unnecessary duplication of trials. Nevertheless, it is essential to ensure the protection of patient data and prevent data leakage or misuse during the data-sharing process. Moving forward, there is a need to establish more robust frameworks for data anonymization and sharing to balance scientific progress with ethical responsibilities.

## 5 Limitations

Our study has several limitations. First, WHO ICTRP serves as a platform integrating trial registries worldwide, yet variations in data field definitions, entry standards, and reporting requirements across different national registries may introduce heterogeneity that could affect the comparability and reliability of pooled analyses. Second, the identification and removal of duplicate records rely primarily on limited common fields. Incomplete or inconsistently reported information in these fields may lead to the erroneous exclusion of valid records or the retention of duplicates, thereby influencing the completeness of the final sample and the statistical power of the analysis. Finally, as registration of observational studies is generally not mandatory, our analysis might have missed relevant investigations, introducing potential selection bias.

## 6 Conclusion

Our comprehensive systematic analysis of MCI trials registered on the WHO ICTRP reveals significant global trends in research activity. The data indicate a marked increase in worldwide trial registrations, and Asian countries contribute the highest volume of studies. Non-pharmacological interventions dominate the therapeutic landscape of registered trials, whereas pharmacological approaches are limited to early-phase clinical development. Emerging hybrid modalities combining traditional cognitive training with computer-assisted technologies show particular promise as a growing research focus, which may become a trend in future investigations. However, these advancements are constrained by persistent deficiencies in data sharing practices and overall trial transparency within the MCI research domain, potentially impeding the reproducibility and clinical translation of these innovative approaches.

## Data Availability

The raw data supporting the conclusions of this article will be made available by the authors, without undue reservation.
